# Clinical practice guideline on undernutrition in chronic kidney disease

**DOI:** 10.1186/s12882-019-1530-8

**Published:** 2019-10-16

**Authors:** Mark Wright, Elizabeth Southcott, Helen MacLaughlin, Stuart Wineberg

**Affiliations:** 1 St James’s University Hospital, Leeds, UK; 2 Kings College Hospital, London, UK; 3 Patient Representative, c/o The Renal Association, Bristol, UK

## Introduction

### Background

“Malnutrition” describes both over and undernutrition. In the UK, the National Institute for Health and Care Excellence (NICE) define malnutrition as “a state in which a deficiency of nutrients such as energy, protein, vitamins and minerals causes measurable adverse effects on body composition, function or clinical outcome” in their Clinical Guideline (CG 32) [1] and Quality Standard (QS24) [2]. These guidelines suggest that patients at high risk of malnutrition should be screened and referred for specialist support if it is present.

Patients with kidney failure face a number of challenges to their nutritional balance [3]. Uraemia itself inhibits appetite and reduces nutrient intake. Dialysis treatments result in the loss of small amounts of nutrients too. There are also other factors that tend to promote protein breakdown over protein synthesis. These include acidosis, insulin resistance and chronic inflammation. This latter factor can coexist with extensive atherosclerotic disease in a particularly toxic combination that is associated with increased mortality risk [3]. The International Society of Renal Nutrition and Metabolism (ISRNM), recommend the term protein energy wasting (PEW) to describe the undernutrition that is prevalent in renal failure populations [4], though more than one underlying cause may present in any individual.

Protein energy wasting is described in 20–40% of patients with stage 4–5 chronic kidney disease (CKD) [3]. It is a more frequent finding in dialysis patients where it is described in 28–54% [5] and is associated with reduced survival, poor healing, infection risk, impaired functional ability and reduced quality of life [3].

### Aim

This clinical practice guideline reviews existing recommendations from International Society for Renal Nutrition & Metabolism, European Society for Parenteral and Enteral Nutrition, Kidney Disease Improving Global Outcomes, American Society for Parenteral and Enteral Nutrition and European Renal Association/European Dialysis & Transplant Association [6–10] and considers recent guidance on advanced kidney failure from NICE [11] with the aim of reducing variation in practice.

### Scope

This guideline has considered how to help adults with CKD stage 4 and 5.

We have not explored the evidence relating to kidney disease in children.

This guideline is intended for health care professionals and people with kidney disease.

### Appraisal of evidence and development of recommendations

The modified GRADE system was used in accordance with the Renal Association’s “Clinical Practice Guideline Development Manual” [12].

There is a two-level grading system for the strength of recommendations.

A **Grade 1** recommendation is a strong recommendation to do (or not do) something, where the benefits clearly outweigh the risks (or vice versa) for most, if not all patients.

A **Grade 2** recommendation is a weaker recommendation, where the risks and benefits are more closely balanced or are more uncertain.

Explicit methodology is used to describe the quality of evidence.

**Grade A** evidence means high-quality evidence that comes from consistent results from well-performed randomised controlled trials, or overwhelming evidence of some other sort (such as well-executed observational studies with very strong effects).

**Grade B** evidence means moderate-quality evidence from randomised trials that suffer from serious flaws in conduct, inconsistency, indirectness, imprecise estimates, reporting bias, or some combination of these limitations, or from other study designs with special strength.

**Grade C** evidence means low-quality evidence from observational studies, or from controlled trials with several very serious limitations.

**Grade D** evidence is based only on case studies or expert opinion.

### Clinical issues covered

We considered how best to identify those at risk of undernutrition and how best to reduce this risk.

We used the HDAS database search tool that is accessible via Health Education England and NICE to identify information sources. This includes a number of search tools including Pub Med, EMBASE, CINAHL and Medline. This was most recently accessed in November 2018. Search terms included, “screening”, “kidney disease”, “nutrition”, “malnutrition”, “vitamin”, and other terms pertinent to dialysis as deemed necessary. We also searched the Cochrane library for relevant systematic reviews.

## Summary of clinical practice guidelines

### Identification of undernutrition in people with kidney disease (guidelines 1.1–1.3)

#### Guideline 1.1 – screening for risk of undernutrition in CKD

We suggest that patients with stages 4–5 CKD should be screened to identify those at risk of undernutrition (2C):

We suggest that screening should be performed (2D);

- On admission then weekly for inpatients
- At clinic review for outpatients with stage 4–5 CKD
- 2–3 monthly for stable haemodialysis patients
- 2–3 monthly for stable peritoneal dialysis patients
- Screening should be performed earlier if their clinical condition changes

#### Guideline 1.2 – diagnosis of undernutrition in people with kidney disease

**1.2.1 -** We suggest that there should be a clear pathway for prompt referral to specialist renal dietitians when risk of undernutrition is identified. This pathway should include locally agreed timescales for formal assessment. (2D)

**1.2.2 -** We suggest that patients should be assessed by a specialist renal dietitian when they begin education about renal replacement treatment and within one month of starting dialysis or changing dialysis modality (2D)

**1.2.3 -** We suggest that formal nutritional assessments are carried out on those identified to be at risk by screening. These diagnostic assessments will typically be performed by specialist renal dietitians with support from the broader multidisciplinary team. (2B)

**Guideline 1.3** - we recommend that in-patients at risk of malnutrition on screening are also considered at risk of refeeding syndrome. (1D)

### Interventions to reduce the prevalence of undernutrition in people with kidney disease (guidelines 2.1–2.6)

#### Guideline 2.1 – dose of small solute removal to prevent anorexia

We recommend that dialysis dose meets recommended solute clearance index guidelines (e.g. URR, Kt/V). (1C)

#### Guideline 2.2 – correction of metabolic acidosis

We suggest that venous bicarbonate concentrations should be maintained in the normal range (2C)

#### Guideline 2.3 – daily dietary protein intake

We recommend a protein intake of:

- 0.8–1.0 g/kg ideal body weight (IBW)/day for patients with stage 4–5 CKD not on dialysis (1C)
- 1.1–1.4 g/kg IBW/day for patients treated on maintenance haemodialysis (1C)
- 1.0–1.2 g/kg IBW/day for patients treated with peritoneal dialysis (1C)
- This should be accompanied by an adequate energy intake. (1C)
- We do not believe there is sufficient evidence to routinely recommend low protein diets for people with progressive kidney disease (1C)

#### Guideline 2.4 – daily energy intake

We suggest a prescribed energy intake of:

- 30–40 kcal/kg IBW/day for all patients depending upon age and physical activity (2C).
- We note that peritoneal dialysis patients are likely to absorb glucose from their dialysis fluid and this should be taken into account.

#### Guideline 2.5 – micronutrient supplementation in patients on dialysis

We suggest that water soluble vitamin supplements should be offered to patients on dialysis with a reduced nutrient intake or those that have unusually high levels of solute clearance on dialysis (e.g. daily or overnight haemodialysis). (2C)

We recommend that other micronutrients are supplemented only if there are symptoms consistent with deficiency and biochemical evidence of deficiency. (1C)

### Interventions to treat undernutrition in people with kidney disease (guidelines 3.1–3.3)

#### Guideline 3.1 anabolic agents in kidney disease

We recommend that anabolic agents should not be used to treat undernutrition in people with kidney disease. (1C)

#### Guideline 3.2 – Oral nutritional supplements in patients who are undernourished

We recommend the use of oral nutritional supplements (ONS) when nutritional intake fails to increase, despite intervention and advice, and remains inadequate to meet energy and protein requirements. (1C)

#### Guideline 3.3 – enteral feeding in patients who are undernourished

We suggest that the use of enteral tube feeding is considered in selected cases if nutrient intake is suboptimal despite oral nutritional support recognising that there are significant risks and inconvenience associated with these forms of feeding (2C). It is important to consider the patient’s comorbidity, general condition and likely survival prospects before initiating enteral tube feeding.

#### Guideline 3.4 – parenteral nutritional support in patients who are undernourished

We suggest intradialytic parenteral nutrition (IDPN) in haemodialysis or intraperitoneal amino acids in peritoneal dialysis may be considered for selected cases when oral or enteral intake is suboptimal (2D).

## Summary of audit measures

**Audit Measure 1:** The service should be able to demonstrate that there is a clear pathway for nutrition care that states how patients at risk of undernutrition will be identified, who they should be referred to and timescales for formal assessment.

**Audit Measure 2**: The service should be able to demonstrate that “at risk” patients were assessed by a specialist renal dietitian within the locally agreed timeframe.

**Audit Measure 3:** The service should be able to demonstrate that all patients commencing dialysis (or changing modality) are assessed by a specialist renal dietitian within four weeks.

## Rationale for clinical practice guidelines

### Identification of undernutrition in people with kidney disease (guidelines 1.1–1.3)

#### Guideline 1.1 – screening for risk of undernutrition in CKD

We suggest that patients with stages 4–5 CKD should be screened to identify those at risk of undernutrition (2C):

We suggest that screening should be performed (2D);

- On admission then weekly for inpatients
- At clinic review for outpatients with stage 4–5 CKD
- 2–3 monthly for stable haemodialysis patients
- 2–3 monthly for stable peritoneal dialysis patients
- Screening should be performed earlier if their clinical condition changes

##### Rationale

Protein energy wasting is common in people with kidney disease. In the whole UK population, malnutrition is estimated to cost £13 billion [13]. It is estimated to cost twice as much to treat patients who are malnourished in hospital than their well-nourished counterparts [14]. This has led to a policy of screening people to see if they are at risk of having malnutrition [15]. Early identification means that some contributing factors can be corrected and progression may be delayed or reversed. That said, research evidence demonstrating improvements in mortality, hospital admissions or length of stay, whether it be in the general population [16] or those with kidney disease, is not strong [17, 18].

1. In the UK, NICE produced a quality standard on nutrition support (QS24) [15]. This was reviewed in 2017 and no changes were introduced. This set out five statements regarding nutritional screening as follows: People in care settings are screened for the risk of malnutrition using a validated screening tool.
2. People who are malnourished or at risk of malnutrition have a management care plan that aims to meet their nutritional requirements.
3. All people who are screened for the risk of malnutrition have their screening results and nutrition support goals (if applicable) documented and communicated in writing within and between settings.
4. People managing their own artificial nutrition support and/or their carers are trained to manage their nutrition delivery system and monitor their wellbeing.
5. People receiving nutrition support are offered a review of the indications, route, risks, benefits and goals of nutrition support at planned intervals

This aims to involve the whole multidisciplinary team and the patient in a conversation about nutrition. Formal assessment of nutrition status is time consuming and requires specific training and expertise. The NICE guidelines suggest that screening, which requires a simple and valid screening tool, is undertaken by health care workers to identify those at risk of malnutrition so that specialist dietitians can focus on those identified as at risk by screening.

The “Malnutrition Universal Screening Tool” (MUST) is recommended by NICE for population screening. This simple system can be taught to non-specialists, but it depends upon changes in weight. People with kidney disease tend to retain fluid as their kidney problem worsens and lose fluid when dialysis starts, so measures that depend upon actual body weight are not as reliable as usual. MUST has poor sensitivity in dialysis patients [19, 20].

The 2018 NICE guidance on renal replacement therapy (NG107) recognises that the relative number of specialist renal dietitians varies between centres [18]. They recommend assessment when patients commence dialysis or choose to pursue a conservative treatment path. They make no further recommendations about undernutrition because they felt that the available evidence lacked strength. This leaves kidney services with apparently conflicting advice.

There has been a lot research into undernutrition in kidney disease. Very few intervention studies of appreciable size look at end points like quality of life, frequency of hospital admission, length of stay in hospital or mortality. This means that it is not possible to confidently predict benefit in these parameters. It does not rule out the possibility of benefit though.

We continue to favour screening for risk of undernutrition in keeping with NICE CG32. There are a number of modified tools described in the literature. Screening tools need to be simple to use and easy to teach to non-specialist staff. They also need to be reproducible and reliable in their ability to find people at risk of having undernutrion [15]. Training materials are useful too. The work by Yamada [20] Campbell [21], Xia [22], Jackson [23]or MacLaughlin [24] may offer a solution for your unit and we recommend that your multidisciplinary team look at these and others to decide how best to screen your patients.

There is little evidence to guide the frequency of screening. NICE recommend that outpatients should be screened at first consultation and in-patients upon admission. They recommend that in-patients should be re-screened weekly. Outpatients with stage 4 or 5 CKD are at high risk for undernutrition and should be screened at each outpatient visit, taking account of changes in fluid balance and recent changes in the amount of food being eaten. People with progressive stage 4 CKD are likely to benefit from education from specialist renal dietitians in terms of advice about energy, salt, potassium and phosphate intake even if they do not flag as a concern on nutritional screening tests [25]. We suggest that established patients on dialysis should be screened every 2–3 months. If concerns arise due to intercurrent illness, screening should be repeated at that time.

#### Guideline 1.2 – diagnosis of undernutrition in people with kidney disease

**1.2.1 -** We suggest that there should be a clear pathway for prompt referral to specialist renal dietitians when risk of undernutrition is identified. This pathway should include locally agreed timescales for formal assessment. (2D).

**1.2.2 -** We suggest that patients should be assessed by a specialist renal dietitian when they begin education about renal replacement treatment and within one month of starting dialysis or changing dialysis modality (2D).

**1.2.3 -** We suggest that formal nutritional assessments are carried out on those identified to be at risk by screening. These diagnostic assessments will typically be performed by specialist renal dietitians with support from the broader multidisciplinary team. (2B).

##### Rationale

Our recommendations aim to ensure that services have clear pathways for referral to specialist renal dietitians. These individuals should have received specialist training in the techniques used to diagnose undernutrition in people with kidney disease.

People deemed to be at risk of undernutrition should be referred for assessment. This uses more sophisticated techniques to determine whether or not undernutrition is present and how severe it is and decide to guide a treatment plan.

ASPEN use the following six evidence based criteria for nutritional assessment:

- Insufficient energy intake
- Weight loss
- Loss of muscle mass
- Loss of subcutaneous fat
- Fluid accumulation that can sometimes mask weight loss
- Diminished “functional status” as measured by hand grip strength.

The presence of two or more of these would lead to a diagnosis of undernutrition.

SGA includes gastrointestinal symptoms (appetite, anorexia, nausea, vomiting, diarrhoea), weight change in the preceding 6 months and last 2 weeks, evidence of functional impairment and a subjective visual assessment of subcutaneous tissue and muscle mass [26].

Modern bioimpedance analysis or spectroscopy devices are primarily designed to assess fluid status but have also been used in nutritional assessment. They require training and expertise, but a wide variety of staff can be taught [27].

Assessment should be carried out by a specialist renal dietitian with knowledge of renal disease and sufficient experience to know which of these tools will be most useful for each individual patient. Medical and nursing staff will also be involved in diagnosing the causes for malnutrition. Social workers, psychologists and community health teams may also have a role to play in individual cases.

Uraemic symptoms will need to be assessed to ensure that they are adequately controlled. If they are not, the reasons for this will need to be determined and rectified [28–31].

Other systemic diseases, especially inflammatory conditions (infection, non-functioning transplants, etc.) and intestinal disease will need to be diagnosed and treated where possible [32].

Dentition may need some attention to make it easier to chew food and address caries & gum disease.

Depression reduces food intake so this may require support or treatment if present [33].

Changes in social circumstances may influence the availability of food.

Some medication can influence appetite and could be reviewed.

Nutritional status is known to deteriorate as chronic disease progresses [34], and is a strong predictor for increased morbidity and mortality. Assessment of nutritional status should therefore be considered when patients begin education for kidney replacement treatment as part of their overall care as well as for potential intervention regarding salt, potassium, phosphate and protein / energy intake assessments [25]. Dietetic assessment is needed at dialysis commencement [18] and if the mode of dialysis changes. This is an important time to assess nutritional status and dietary knowledge in terms of potassium, phosphate, salt and fluid. It is also a good time to reassess the patient’s individualized nutritional care plan, prioritising which bits of information are most important for each person. More than one visit is likely to be needed. The advice given may change over time depending upon the response to dialysis and other changing circumstances.

For stable patients, nutritional changes are likely to be gradual after this [28]. Screening should help to remind both front line staff and patients that nutritional status can change quickly. If there are concerns about weight loss, changes in physical appearance or reported reduced food intake between screening cycles, screening should be repeated earlier or advice sought from specialist renal dietitians. The importance of multidisciplinary working between doctors, nurses and specialist renal dietitians in this regard cannot be over-emphasised.

#### Guideline 1.3 - we recommend that in-patients at risk of malnutrition on screening are also considered at risk of refeeding syndrome. (1D)

##### Rationale

The criteria used to identify those at risk in accordance with NICE guideline CG 32 [19] can be located, along with the management of refeeding syndrome on the British Association of Parenteral and Enteral Nutrition website on the following link:

www.bapen.org.uk/nutrition-support/assessment-and-planning/nutritional-assessment?start=2

Refeeding problems include life threatening acute micronutrient deficiencies, fluid and electrolyte imbalances and disturbances of metabolic processes which result from over-rapid or unbalanced nutrition support [35]. Patients at risk of refeeding syndrome require prompt assessment by dietitians and careful multidisciplinary working.

Biochemical changes that can occur as a result of refeeding include hypophosphataemia, hypokalaemia, hypomagnesaemia and occasionally hypocalcaemia. Acute clinical conditions can result from this including cardiac failure, pulmonary oedema and dysrhythmia, acute circulatory fluid overload or fluid depletion and deterioration in neurological state and confusion. When assessing biochemical markers, use pre-dialysis bloods to avoid over-supplementation.

Thiamine is an essential coenzyme for carbohydrate metabolism, if depleted due to inadequate intakes/starvation this can lead to Wernicke’s encephalopathy or Korsakoff’s syndrome. Replacement of thiamine and other B vitamins is an important component of managing risk of refeeding syndrome. The link above also gives recommendations for vitamin replacement regimes.

### Interventions to reduce the prevalence of undernutrition in people with kidney disease (guidelines 2.1–2.6)

#### Guideline 2.1 – dose of small solute removal to prevent anorexia

We recommend that dialysis dose meets recommended solute clearance index guidelines (e.g. URR, Kt/V).(1C)

##### Rationale

Dialysis treatment to current Kt/V or URR standards is associated with better nutrient intake than lower doses. Attempts to increase the small solute clearance further have not demonstrated progressive improvement.

Current recommendations can be accessed here: https://renal.org/guidelines/

#### Guideline 2.2 – correction of metabolic acidosis

We suggest that venous bicarbonate concentrations should be maintained in the normal range (2C)

##### Rationale

Acidosis is an established catabolic factor [36] and the bicarbonate concentration of patients on peritoneal dialysis and HD patients should be maintained within target range to minimise this [37]. Recent data suggests that predialysis serum bicarbonate levels of > 28 mmol/l may be associated with adverse outcomes [38]. Bicarbonate supplementation in the low clearance clinic may retard the progression of kidney disease [39].

#### Guideline 2.3 – daily dietary protein intake

We recommend a minimum protein intake of:

- 0.8–1.0 g/kg ideal body weight (IBW)/day for patients with stage 4–5 CKD not on dialysis (1C)
- 1.1 g/kg IBW/day for patients treated with haemodialysis (1C)
- 1.0–1.2 g/kg IBW/day for patients treated with peritoneal dialysis (1C)
- This should be accompanied by an adequate energy intake. (1C)
- We do not believe there is sufficient evidence to recommend low protein diets for people with progressive kidney failure (1C)

##### Rationale

Before dialysis was widely available, advanced kidney disease was treated by greatly restricting protein intake. This reduced the generation of uraemic toxins. In theory, it could slow the rate of damage to kidneys as well [40]. There has been interest in using this approach to delay the point at which dialysis begins. Conversely, there has also been concern that such an approach is likely to lead to malnutrition with the associated loss of function and poor quality of life. A Cochrane review looked at 17 studies including 2996 adults without diabetes [41]. They concluded that very low protein diets (0.3–0.4 g/kg/day) may delay the point at which dialysis is needed. More modest protein restriction (0.5–0.6 g/kg/day) does not have an effect. They observed that there wasn’t enough information available to confirm that quality of life is maintained on very low protein diets. Concordance with these diets was difficult.

A separate Cochrane review from 2007 looked at protein restriction in diabetic adults [42]. This did not find sufficient evidence to recommend protein restriction, though they noted that the low protein diet groups had not restricted their intake to the prescribed level in the study protocols.

The recommended protein intakes for dialysis patients are derived from a literature review carried out in 2013 [43] and a systematic review published in 2014 [44]. Both recognise that the evidence is not strong. Protein intakes less than 0.8 g/kg and more than 1.4 g/kg have been associated with increased risk of mortality [43, 45], In the presence of inter-current illness protein requirements may be higher. Recent recommendations for patients with acute kidney injury and intercurrent illness can be accessed at:

https://www.thinkkidneys.nhs.uk/aki/wp-content/uploads/sites/2/2017/12/Think-Kidneys-Nutrition-Guide-final.pdf

An adequate energy intake is needed to ensure that consumed protein is used effectively by the body [46].

#### Guideline 2.4 – daily energy intake

We suggest a prescribed energy intake of

- 30–40 kcal/kg IBW/day for all patients depending upon age and physical activity (2C).
- We note that peritoneal dialysis patients are likely to absorb glucose from their dialysis fluid and this should be taken into account.

##### Rationale

The recommended energy intake is consistent with other guidelines. As above, it is important that this energy intake is achieved to ensure that dietary protein is used effectively. We have not identified any new evidence that this should change. An individual’s recommended intake will depend upon their age, gender, level of physical activity and intercurrent medical issues. Peritoneal dialysis patients absorb some glucose from their dialysis fluid. This will vary depending upon their dialysis regime and solute transporter status. The amount of absorption can be quantified by measuring effluent volume and glucose concentration and comparing this with the total glucose content of the prescribed fluid regime that day [47].

#### Guideline 2.5 – micronutrient supplementation in patients on dialysis

We suggest that water soluble vitamin supplements should be offered to dialysis patients with a reduced nutrient intake or those that have unusually high levels of solute clearance on dialysis (e.g. daily or overnight haemodialysis). (2C)

We recommend that other micronutrients are supplemented only if there are symptoms consistent with deficiency and biochemical evidence of deficiency. (1C)

##### Rationale

Haemodialysis removes water soluble vitamins from the circulation. Data from the DOPPS database suggested that supplementation of water soluble vitamins was associated with significantly lower mortality rates at patient and institution level [48]. A subsequent systematic review gathered information on vitamin levels in (mostly) American patients on haemodialysis [49]. They found that there was some evidence of low thiamine levels in some studies and vitamin B6 levels can also be low. Other water soluble vitamins were usually present in normal levels or there was no data. The authors caution that there may be differences between nations given that different countries fortify foods in different ways. Most of the source data was old. Dialysis patterns have changed during the intervening years. It is possible that there is greater removal of vitamins with modern treatment regimes.

A recent review gathered more up to date information on dialytic removal of micronutrients in haemodialysis [50] and peritoneal dialysis [51]. A very recent case study demonstrated that depletion of water soluble vitamins is a real risk in this group of people, although it is rare [52].

We suggest that supplementation should not be offered routinely, but it should be considered if nutritional assessment suggests that dietary intake of vitamins is likely to be low or dialysis dose is unusually high. The European Best Practice Guidelines cite recommended intake levels for the various water soluble vitamins. There is no reason to routinely supplement trace elements such as zinc or selenium [53]. Supplements should be considered if there are symptoms consistent with deficiency and low blood levels.

### Interventions to treat undernutrition in people with kidney disease (guidelines 3.1–3.4)

#### Guideline 3.1 - anabolic agents in kidney disease

We recommend that anabolic agents should not be used to treat undernutrition in people with kidney disease. (1C)

##### Rationale

These have been used in small studies. A large study using Growth Hormone terminated early. It did not demonstrate benefit in mortality or quality of life [54]. Anabolic steroids have significant serious side effects which greatly limit their potential for use [55].

#### Guideline 3.2 Oral nutritional supplements in patients who are undernourished

We recommend the use of oral nutritional supplements (ONS) when nutritional intake fails to increase, despite intervention and advice, and remains inadequate to meet energy and protein requirements. (1C)

##### Rationale

Nutrition support should be implemented to reverse diagnosed malnutrition and should involve ‘food first’ by the means of high energy high protein snacks, little and often approach to eating and food fortification [56] The nutritional intake of patients on haemodialysis was improved with intervention from a specialist renal dietitian [57], as was the patients’ quality of life [58]. A systematic review from 2005 included eighteen studies, of which five were randomised controlled trials [59]. It found that ONS improved serum albumin and dietary intakes but there was inadequate evidence to show improved outcomes such as quality of life and mortality. Oral nutritional supplements have been found to reduce hospitalisation [60]. A more recent systematic review also found oral nutritional support improved some nutritional markers [61].

There have been recent publications to support the routine prescription of ONS treatment in patients on haemodialysis [60–63] and peritoneal dialysis [64]. They imply that patients engaging with dietetic support programs do better than those that don’t. There have been several studies looking at intradialytic oral nutrition, whereby nutritional supplements are consumed on haemodialysis. This method of nutrition support has been associated with reduced hospitalisation rates and mortality risk [65, 66].

#### Guideline 3.3 - enteral feeding in patients who are undernourished

We suggest that the use of enteral feeding is considered in selected cases if nutrient intake is suboptimal despite ONS recognising that there are significant risks and inconvenience associated with these forms of feeding (2C). It is important to also consider the patient’s comorbidity, general condition and likely survival prospects before initiating enteral feeding.

##### Rationale

The evidence to support enteral tube feeding is limited to a few small observational studies [67, 68]. A review of 181,196 percutaneous endoscopic gastrostomy (PEG) insertion procedures in the US found that patients with CKD had a 1.6-fold increased risk of mortality [69]. Having malnutrition increased mortality risk to 5.25-fold when compared to patients with head and neck disease preventing oral intake. It is important to note that the review did not state the stage of kidney disease, if the patients were receiving renal replacement therapy or how they defined malnutrition.

#### Guideline 3.4 - parenteral nutritional support in patients who are undernourished

We suggest intradialytic parenteral nutrition (IDPN) in haemodialysis or intraperitoneal amino acids in peritoneal dialysis may be considered for selected cases when oral or enteral intake is suboptimal (2D).

Intradialytic parenteral nutrition (IDPN) can be used as a form of nutrition support in maintenance patients on haemodialysis. The FINE study [70] compared ONS to ONS and IDPN in a randomised controlled trial. Survival rates did not differ between the two groups, and both groups improved nutritional markers. The study did not report data on quality of life, hospital admission rates or length of stay. A subsequent systematic review set out to identify if IDPN improved survival, quality of life, or nutritional status [71]. It concluded that there was insufficient evidence to demonstrate benefit or harm with the use of IDPN in malnourished haemodialysis patients. A more recent randomised controlled trial found a change in a biochemical marker but no improvement in quality of life scores or SGA rating [72]. Despite the lack of evidence for widespread use of IDPN, it may be helpful in a small number of selected cases when other options have been exhausted.

Use of “total” parenteral nutrition is recommended as per BAPEN/ESPEN guidelines when the gut is non-functioning or inaccessible.

Intraperitoneal amino acid (IPAA) solutions use 1.1% amino acid-based solution in place of a dextrose-based peritoneal dialysate. Studies that have shown IPAA with bicarbonate buffered fluid increase protein synthesis [73]. It appears that having the amino acid solution administered in a fed state helps to increase protein synthesis [74]. The studies were short in duration and failed to see a significant improvement in nitrogen balance. In a 3-year, randomized controlled study, 60 malnourished CAPD patients were randomly assigned to either replace one daily exchange with IPAA or to continue with dextrose dialysate [75]. Dietary protein intake increased in the IPAA group. Biochemical nutritional parameters stayed stable or increased in the IPAA group but decreased in the dextrose group. There was no effect on patient survival. As such, this product may have a place in the treatment of a small number of selected individuals.

## Lay summary

Undernutrition is common in people with kidney disease. It is linked to problems that can affect quality of life and wellbeing. Based on the research that has been done, the risk of not being able to eat enough food to maintain usual weight is higher in people with kidney disease than in those without kidney disease.

We recommend that all people with advanced kidney disease have access to a specialist renal dietitian to help understand more about how to eat healthily. Diet affects several aspects of kidney disease and it can be tricky to find a balance between restricting things that can cause problems and taking enough of the things that are needed.

Kidney services should be able to look out for signs that undernutrition is developing and have a detailed plan about what to do should it be identified. If it looks as though this is happening, those affected should be given the opportunity to talk to a dietitian who is qualified to treat undernutrition in people with kidney disease.

Sometimes, there can be more than one reason for undernutrition, so several different specialists might need to work together to help. When someone cannot eat enough ordinary food to stay well nourished, supplements or other forms of additional nutrition may be recommended.

It appears that regular exercise is good for people having dialysis. It can help to preserve muscle function and makes people feel better.
